# Supplementary material for: Direct synthesis of ordered mesoporous materials from thermoplastic elastomers
Source: Nat Commun. 2023 Feb 6;14:639. doi: 10.1038/s41467-023-36362-x (PMC9902477; doi:10.1038/s41467-023-36362-x)
Supplement: Supplementary file 1 — Supplementary Information [file 41467_2023_36362_MOESM1_ESM.pdf]

Supplementary Information

Direct synthesis of ordered mesoporous materials from thermoplastic elastomers

Mark Robertson, Alejandro Guillen-Obando, Andrew Barbour, Paul Smith, Anthony Griffin, Zhe Qiang*

School of Polymer Science and Engineering, University of Southern Mississippi, Hattiesburg, 39406, Mississippi, USA

Corresponding Author: Zhe Qiang: zhe.qiang@usm.edu

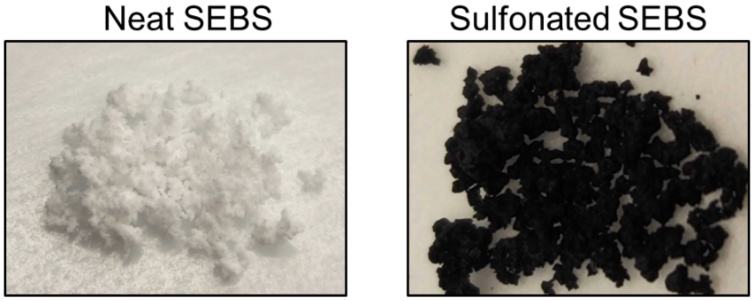

Supplementary Figure 1. Visual demonstration of the structural retention of the SEBS118 powders throughout the crosslinking process.

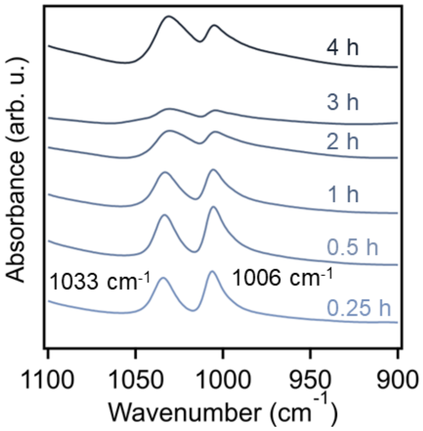

Supplementary Figure 2. FTIR spectra of SEBS118 as a function of sulfonation time, indicating the peaks associated with reaction with the poly(ethylene-*ran*-butylene) and polystyrene blocks of the BCP.

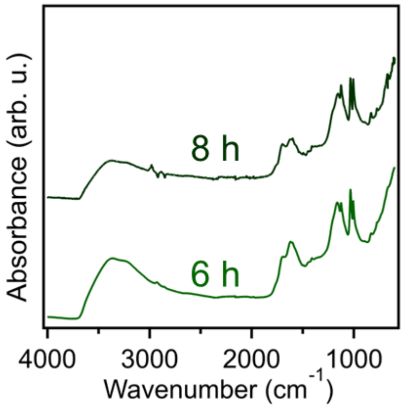

Supplementary Figure 3. FTIR spectra of SEBS118 after 6 h and 8 h of crosslinking time.

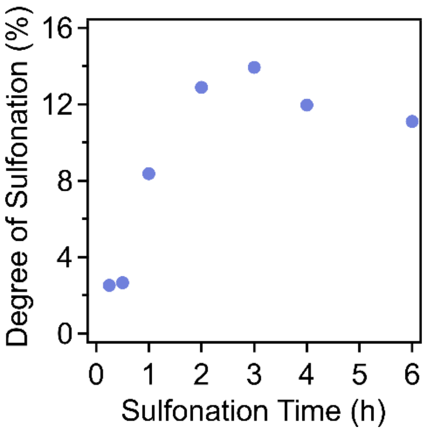

Supplementary Figure 4. Degree of sulfonation determined through Mohr's titrations of the crosslinked material as a function of reaction time.

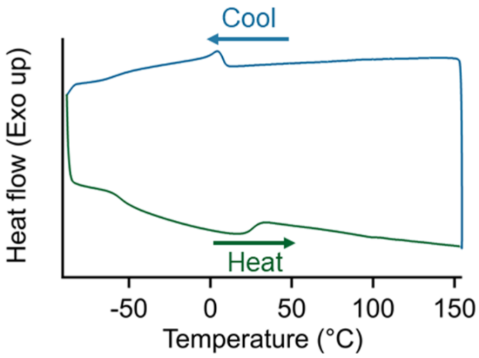

Supplementary Figure 5. Differential scanning calorimetry of thermogram of neat SEBS118 TPE. The cool and second heat cycles demonstrate completely amorphous material with no melting or crystallization peaks.

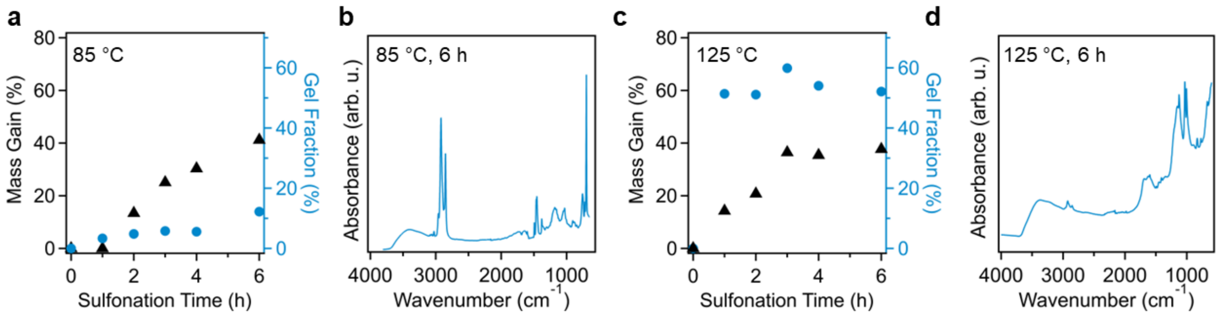

Supplementary Figure 6. (a) Mass gain and gel fraction of SEBS118 sulfonated at 85 °C as a function of sulfonation time and (b) FTIR spectrum of SEBS118 sulfonated at 85 °C for 6 h. (c) Mass gain/gel fraction of SEBS118 sulfonated at 125 °C as a function of reaction time and (d) FTIR spectrum of SEBS118 reacted at 125 °C for 12 h.

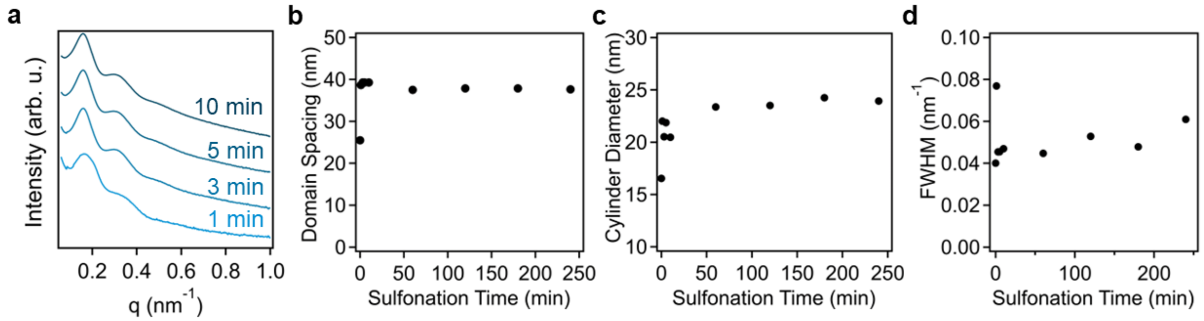

Supplementary Figure 7. (a) SAXS patterns of sulfonated SEBS118 at short time scales. (b) Domain spacing determined from the primary peak in SAXS patterns as a function of reaction time. (c) Cylinder diameter determined from fitting SAXS patterns with scattering functions that include a flexible cylinder form factor which represents the size and shape of the cylindrical minority PS domains within the polymer. (d) FWHM determined from the primary peaks within the SAXS patterns to demonstrate the evolution of degree of ordering throughout the reaction process.

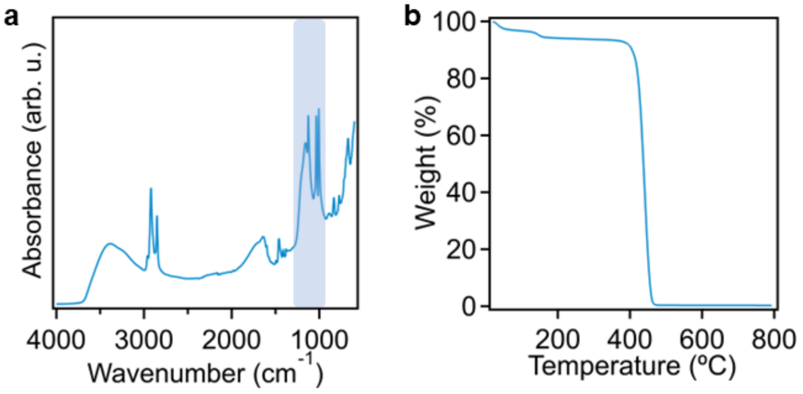

Supplementary Figure 8. (a) FTIR spectrum of SEBS118 sulfonated for 12 h at 85 °C. (b) TGA thermogram of SEBS118 sulfonated for 12 h at 85 °C.

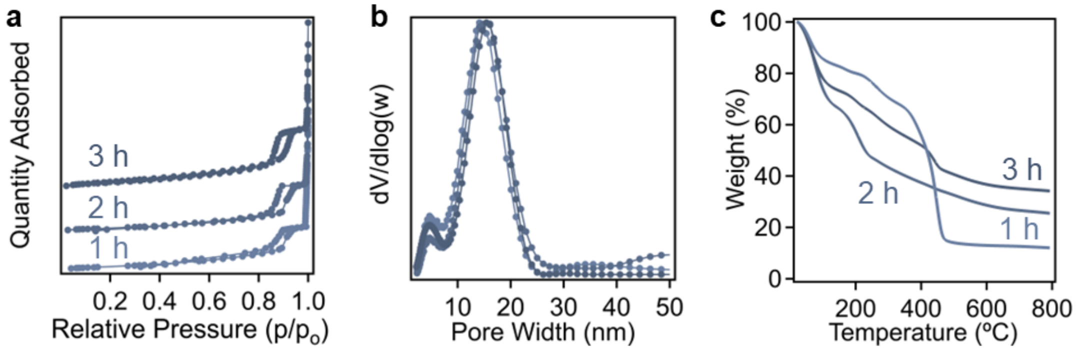

Supplementary Figure 9. (a) Nitrogen physisorption isotherms and (b) pore size distributions of SEBS118-derived OMC which were sulfonated for 1 h, 2 h, and 3 h. (c) TGA thermograms of sulfonated SEBS118 illustrating the dependence of carbon yield on reaction time.

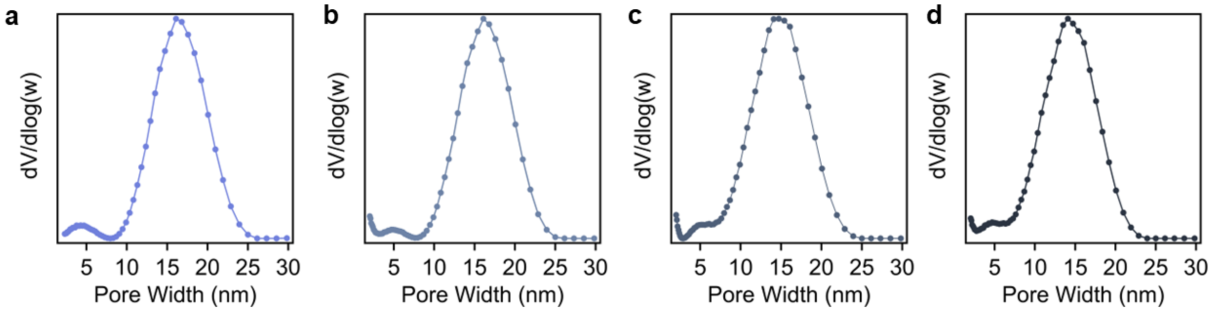

Supplementary Figure 10. Pore size distributions determined using nitrogen physisorption and NLDFT models for carbon slit pores at 77 K for porous materials calcinated and carbonized at (a) 400 °C, (b) 800 °C, (c) 1000 °C, and (d) 1200 °C, respectively.

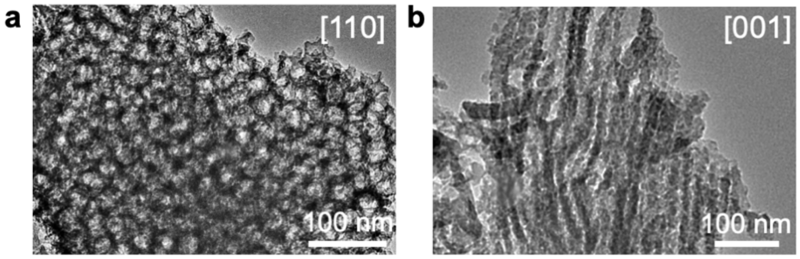

Supplementary Figure 11. TEM images of SEBS118-800 along the (a) [110] and (b) [001] directions.

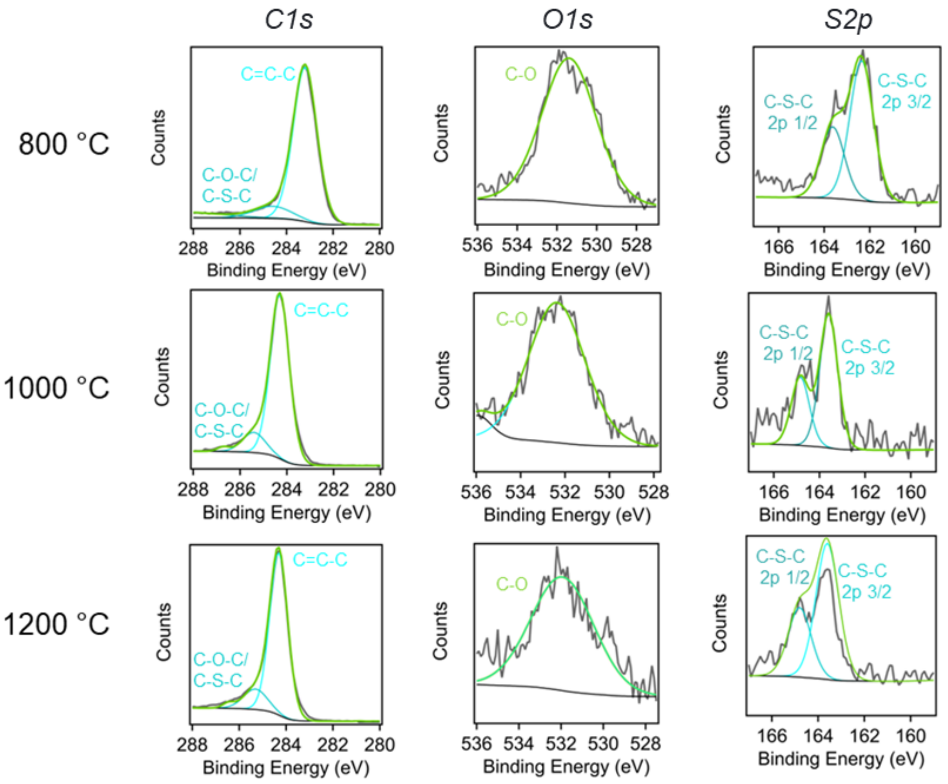

Supplementary Figure 12. High resolution XPS scans of *C1s*, *O1s*, and *S2p* for SEBS118-derived OMC carbonized at 800 °C, 1000 °C, and 1200 °C.

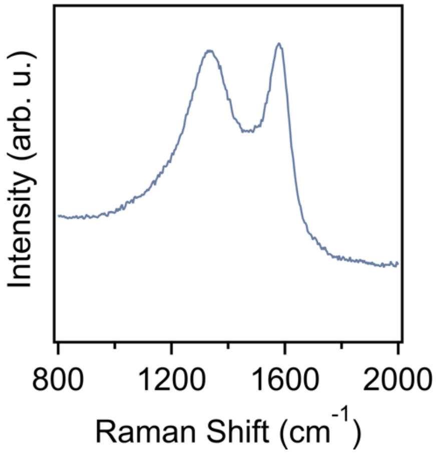

Supplementary Figure 13. Raman spectra for SEBS118-OMC carbonized at 800 °C

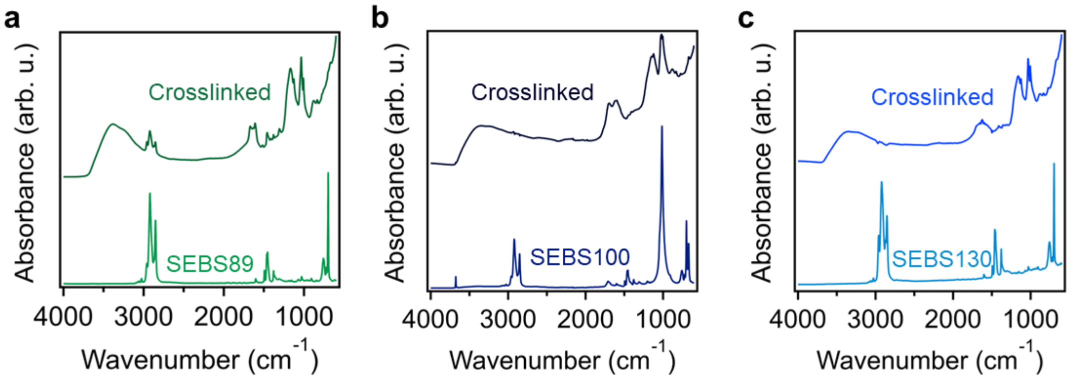

Supplementary Figure 14. FTIR spectra of (a) SEBS89, (b) SEBS100, and (c) SEBS130 before and after crosslinking for 4 h.

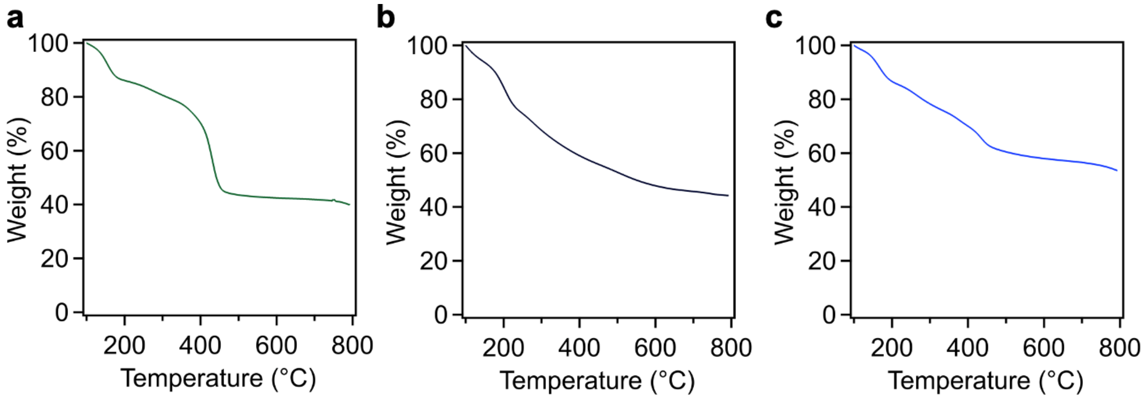

Supplementary Figure 15. TGA thermograms for crosslinked SEBS prepared from (a) SEBS89, (b) SEBS100 and (c) SEBS130.

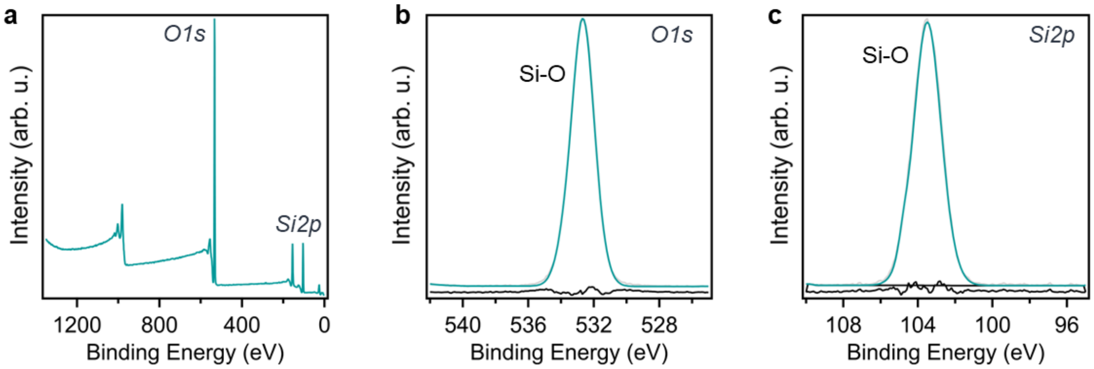

Supplementary Figure 16. (a) XPS survey scan of OMS templated from SEBS118-800. High resolution scans for (b) *O1s* and (c) *Si2p* of the same sample.

Material	Domain Spacing (nm)	Surface Area (m^2/g)	Average Pore Width (nm)	Pore Volume (cm^3/g)	Sulfur Content (at%)
SEBS118-400	32.7	133	16.1	0.20	1.8
SEBS118-800	33.9	357	16.1	0.41	1.5
SEBS118-1000	29.4	404	14.7	0.38	0.9
SEBS118-1200	27.9	212	14.1	0.42	0.7
SEBS89-800	24.2	216	10.4	0.36	0.3
SEBS130-800	24.9	501	4.7	1.33	1.6
SEBS100-800	21.8	475	11.3	0.33	-
SEBS118-OMS	24.9	343	14.7	0.61	0

Supplementary Table 1. Domain spacings, pore textures and sulfur content of SEBS derived mesoporous materials determined through SAXS, nitrogen adsorption/desorption isotherms and XPS, respectively. Samples are named using the following naming convention which consists of SEBSX-Y, where X represents the identity of the polymer precursor and Y represents the calcination/carbonization temperature. SEBS118-OMS is an exception and represents ordered mesoporous silica produced using SEBS118-800 as a template.
